# Identification of the regulatory role of lncRNA HCG18 in myasthenia gravis by integrated bioinformatics and experimental analyses

**DOI:** 10.1186/s12967-021-03138-0

**Published:** 2021-11-18

**Authors:** Shuang Li, Xu Wang, Tianfeng Wang, Huixue Zhang, Xiaoyu Lu, Li Liu, Lifang Li, Chunrui Bo, Xiaotong Kong, Si Xu, Shangwei Ning, Jianjian Wang, Lihua Wang

**Affiliations:** 1 Department of Neurology, The Second Affiliated Hospital of Harbin Medical University, Harbin, 150081 Heilongjiang China; 2 College of Bioinformatics Science and Technology, Harbin Medical University, Harbin, 150081 Heilongjiang China

**Keywords:** Myasthenia gravis, HCG18, miR-145-5p, CD28, Network analysis, Random walk with restart

## Abstract

**Background:**

Long non-coding RNAs (lncRNAs), functioning as competing endogenous RNAs (ceRNAs), have been reported to play important roles in the pathogenesis of autoimmune diseases. However, little is known about the regulatory roles of lncRNAs underlying the mechanism of myasthenia gravis (MG). The aim of the present study was to explore the roles of lncRNAs as ceRNAs associated with the progression of MG.

**Methods:**

MG risk genes and miRNAs were obtained from public databases. Protein–protein interaction (PPI) network analysis and module analysis were performed. A lncRNA-mediated module-associated ceRNA (LMMAC) network, which integrated risk genes in modules, risk miRNAs and predicted lncRNAs, was constructed to systematically explore the regulatory roles of lncRNAs in MG. Through performing random walk with restart on the network, HCG18/miR-145-5p/CD28 ceRNA axis was found to play important roles in MG, potentially. The expression of HCG18 in MG patients was detected using RT-PCR. The effects of HCG18 knockdown on cell proliferation and apoptosis were determined by CCK-8 assay and flow cytometry. The interactions among HCG18, miR-145-5p and CD28 were explored by luciferase assay, RT-PCR and western blot assay.

**Results:**

Based on PPI network, we identified 9 modules. Functional enrichment analyses revealed these modules were enriched in immune-related signaling pathways. We then constructed LMMAC network, containing 25 genes, 50 miRNAs, and 64 lncRNAs. Through bioinformatics algorithm, we found lncRNA HCG18 as a ceRNA, might play important roles in MG. Further experiments indicated that HCG18 was overexpressed in MG patients and was a target of miR-145-5p. Functional assays illustrated that HCG18 suppressed Jurkat cell apoptosis and promoted cell proliferation. Mechanistically, knockdown of HCG18 inhibited the CD28 mRNA and protein expression levels in Jurkat cells, while miR-145-5p inhibitor blocked the reduction of CD28 expression induced by HCG18 suppression.

**Conclusion:**

We have reported a novel HCG18/miR-145-5p/CD28 ceRNA axis in MG. Our findings will contribute to a deeper understanding of the molecular mechanism of and provide a novel potential therapeutic target for MG.

**Supplementary Information:**

The online version contains supplementary material available at 10.1186/s12967-021-03138-0.

## Introduction

Myasthenia gravis (MG) is a chronic acquired autoimmune disease characterized by fluctuating muscular weakness and abnormal fatigability, which is mainly caused by antibodies against acetylcholine receptor (AChR) [1]. Antibodies against muscle‐specific kinase (MuSK) and low‐density lipoprotein receptor‐related protein 4 (LRP4) are also detectable in a small group of MG patients [2]. Current drug treatment strategies for MG mainly include cholinesterase inhibitors and immune-suppressants [3]. However, various side effects are major obstacles to the effective use of these drugs. The rise of nanomedicine has developed a novel era for the treatment of various diseases [4, 5], which provides a potential treatment strategy for MG patients. Fully understanding the mechanism of MG is the basis for effective implementation of nanomedicine. The pathogenesis of MG is complex and involves many factors, such as environment, immunology and genes. Some important genes have been identified to participate in the occurrence and development of MG. For example, Cytotoxic T-lymphocyteantigen-4 (CTLA-4) is a homologue to CD28, the polymorphism of which is related to the increasing susceptibility in MG patients [6]. As known, disease risk genes exert their functions through forming a biological network, other than single gene. Module structure, a densely connected subnetwork, is a common property of multiple different types of networks [7]. The genes in the same module are more likely to play similar roles or share some common properties. In other words, we can infer the functions of the unknown genes according to the other genes’ function in the same module. Hence, it is crucially important to identify functional modules in order to better understand the pathogenesis of MG.

In recent years, the roles of non-coding RNA (ncRNA) in the development of MG have been gradually recognized. Long ncRNAs (lncRNAs) and miRNA, two common types of ncRNA, can regulate the expression of protein-coding genes via multiple different mechanisms [8, 9], which exert important functions in many diseases including MG. For example, aberrantly expressed IFNG-AS1 in MG patients effects CD4^+^ T cell activation by modulating the expression human leukocyte antigen (HLA)-DRB1 expression [10]. Downregulation of miR-181a in the peripheral blood mononuclear cells (PBMCs) of MG patients can affect the production of anti-AChR antibodies and CD4^+^ T cell differentiation by regulating interleukin (IL)-2 expression, which demonstrates that miR-181a contributes to the pathogenesis of MG [11]. miR-653 is decreased in MG mice and inhibits thymocyte proliferation and induces thymocyte apoptosis through suppressing tripartite motif 9 (TRIM9) [12]. Though some lncRNAs and miRNAs have been detected to be correlated to MG, few researchers have focused on the interactions among lncRNAs, miRNAs and genes underlying the mechanism of MG.

Competing endogenous RNA (ceRNA), a new regulatory mechanism between coding RNA and ncRNA, is defined as RNA molecules compete for a common miRNA with other RNAs through miRNA response elements (MREs) [13]. Accumulating evidence demonstrates that lncRNAs as ceRNAs can compete for MREs with diseases risk genes and further participate in the occurrence and development of diseases. For example, lncRNA LINC01133 played a crucial role in the progression and metastasis of gastric cancer by acting as a ceRNA of miR-106a-3p to regulate adenomatous polyposis coli (APC) expression [14]. In addition, lncRNAs as ceRNAs also play important roles in the pathogenesis of autoimmune diseases. lncRNA LOC100912373 regulates the expression of PDK1 by sponging miR-17-5p to promote proliferation of fibroblast-like synoviocytes in rheumatoid arthritis [15]. However, the potential role of ceRNAs in MG remains unclear. Considering the importance of functional modules, there is a need to combine the modules with ceRNA to explore lncRNAs regulatory mechanism in MG and to identify new biomarkers for the diagnosis and treatment for MG.

**Fig. 1:**
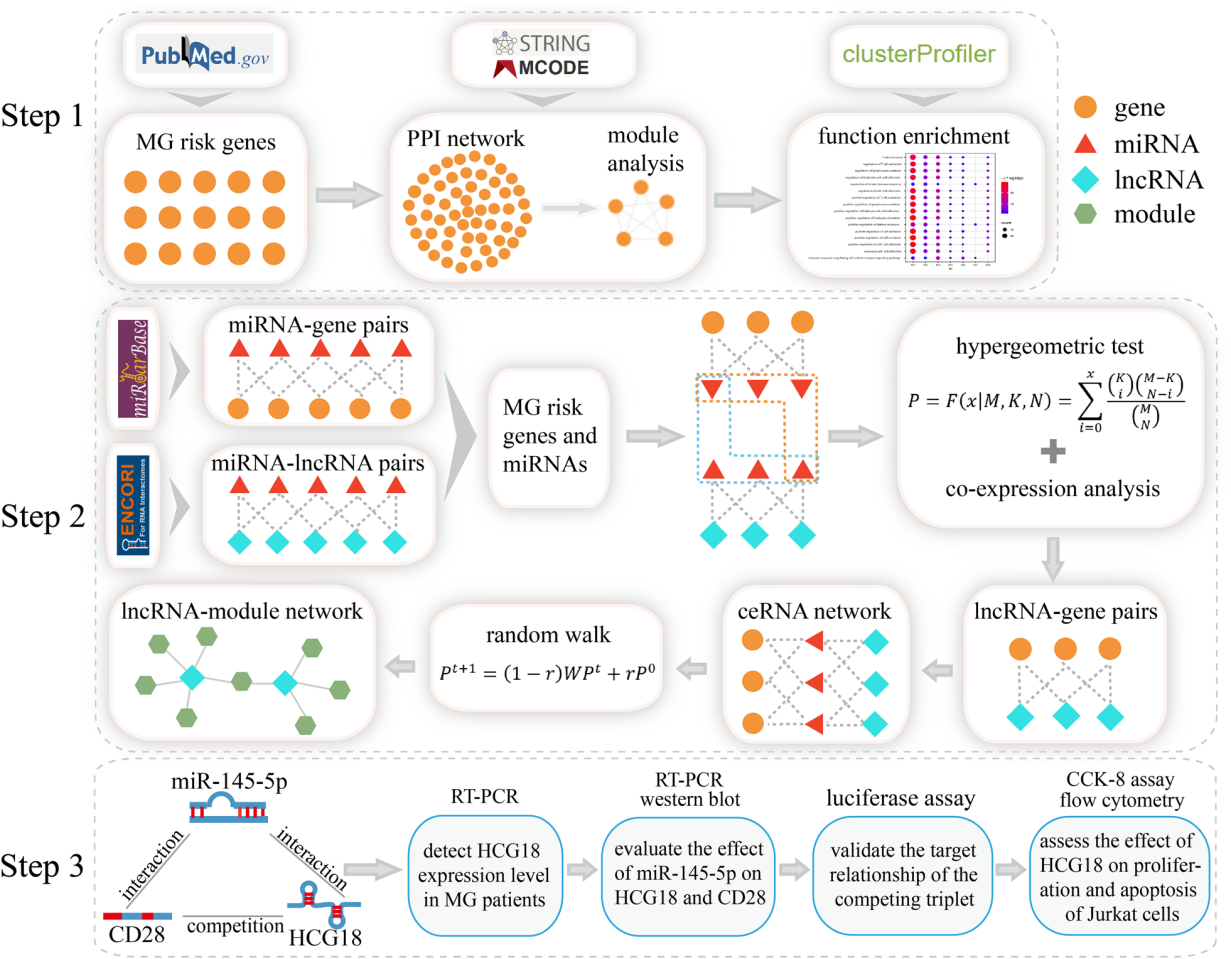
Flowchart in this study. Step 1: Identification of functional modules. Step 2: Excavation of key lncRNAs by a comprehensive computational approach. Step 3: Validation of HCG18 expression and function in MG

To address this point, we designed an integrative analysis pipeline (Fig. 1). We first identified 9 modules by constructing a PPI network using MG risk genes. Functional enrichment analysis was performed to uncover the potential roles of each module. We then constructed a lncRNA-mediated module-associated ceRNA network. Through reliable algorithm and publications, biological experiments were designed to further confirm the presence of an HCG18-mediated ceRNA regulatory mechanism through the miR-145-5p/CD28 axis in MG. Our study revealed potential roles of HCG18 and provided new insights into the exploration of ceRNA regulatory mechanism in MG.

## Materials and methods

### Acquisition of human MG risk genes and miRNAs

To ensure a high quality, the data of MG risk genes was obtained following previously described criteria [16, 17]. First, we obtained all publications from PubMed database using the keyword “myasthenia gravis” to collect experimentally supported MG-related genes. We then manually curated MG-related genes following the criteria we set: (1) the gene was notably differentially expressed in at least five human MG samples and was detected using reliable low-throughput experiment methods, such as RT-PCR and northern blot; or (2) the gene was contained in several current public databases we mentioned previously [16, 17]. In addition, we updated the list of MG risk miRNAs referred to our previous studies published online [18].

### Analysis of PPI and module

Based on the Search Tool for the Retrieval of Interacting Genes (STRING) database [19], PPI network of MG risk genes was established with a confidence score ≥ 0.4 and visualized by means of Cytoscape software. Furthermore, Plug-in Molecular Complex Detection (MCODE) [20] was utilized to identify functionally related and highly interconnected modules from the PPI network with a degree cutoff = 2, node score cutoff = 0.2, k-core = 2 and maximum depth = 100.

### Functional enrichment analysis

To screen the potential functions of modules in the PPI network, gene ontology (GO) functions and KEGG pathway enrichment analysis were performed by clusterProfiler package [21], which is an R package of Bioconductor and can perform statistical analysis and visualization of functional clustering of gene sets or gene clusters. P-value < 0.05 was considered to be significantly enriched function annotations.

### Identification of miRNA-gene and miRNA-lncRNA interactions

First, the interactions of miRNA-gene were acquired from miRTarBase (release 8.0) [22], which is a database that curates experimentally validated miRNA-gene interactions. miRNA-gene pairs were selected when they were supported by western blot and/or reporter assay showing high-confidence functional. Further, the gene and miRNA in each miRNA-gene pair were filtered by aforementioned human MG risk genes of modules and MG risk miRNAs. Ultimately, we only retained the intersections among human MG risk genes in modules, human MG risk miRNAs and experimentally supported miRNA-gene interactions as the candidate miRNA-gene pairs. Then, the interactions of MG risk miRNA and lncRNA were extracted from starBase (v3.0) [23], which is a database decoding the interactions of miRNAs, lncRNAs, mRNAs from large-scale CLIP-Seq data, such as HITS-CLIP, iCLIP, PAR-CLIP, and CLASH.

### Identification of potential gene-lncRNA competing pairs

Hypergeometric test and the Pearson correlation coefficient (PCC) were performed to identify the potential competing gene-lncRNA pairs. First, hypergeometric test was applied for assessing the significance of the shared miRNAs between each gene and lncRNA. The formula is as follows:

$$P=F\left(x|M,K,N\right)=\sum_{i=0}^{x}\frac{\left(\genfrac{}{}{0pt}{}{K}{i}\right)\left(\genfrac{}{}{0pt}{}{M-K}{N-i}\right)}{\left(\genfrac{}{}{0pt}{}{M}{N}\right)}$$

For each gene-lncRNA pair, *M* was the total number of MG risk miRNAs, *K* was the number of miRNAs interacting with one gene, *N* was the number of miRNAs interacting with one lncRNA, and *x* was the number of common shared miRNAs between the gene and the lncRNA. The gene-lncRNA pairs with *P*-value < 0.01 were considered as statistical significance.

We further evaluated the co-expression association of gene-lncRNA pairs obtained above by performing the PCC. We downloaded the expression data of the genes and lncRNAs from the Genotype-Tissue Expression (GTEx, v8 release) project (https://gtexportal.org/home/datasets) [24]. We adjusted the *P*-value of co-expression analysis using the false discovery rate (FDR). The gene-lncRNA pairs with PCC > 0.7 and FDR < 0.01 were finally considered significant gene-lncRNA pairs.

### Construction of the lncRNA-mediated module-associated ceRNA network

Based on the theory that lncRNA shares common miRNA-binding sites with gene and functions as miRNA’s sponge to regulate gene, LMMAC network was constructed. For a given gene-miRNA-lncRNA interaction, both gene and lncRNA that shared common miRNAs were co-expression for merging into a competing triplet. After integrating all of the gene-miRNA-lncRNA competing triplets, we constructed the LMMAC network and visualized using Cytoscape software. In the network, nodes denoted genes, miRNAs, and lncRNAs, and edges denoted their interactions.

### Random walk with restart to prioritize lncRNAs for MG

We used random walk with restart (RWR) [25, 26] to prioritize potential lncRNAs in the LMMAC network for MG by simulating a random walker and randomly moving from a set of source nodes to its network neighbors. It is defined as follows:

$${P}^{t+1}=\left(1-r\right){WP}^{t}+{rP}^{0}$$

where *P*^0^ is the initial probability vector, which is constructed so that a value of 1 was assigned to nodes representing genes known to be associated with disease while 0 was assigned to other nodes; *W* is the column-normalized adjacency matrix of the LMMAC network; *r* is the restart probability at each step of the random walk at source nodes; and *P*^*t*^ is a vector in which the ith element has the probability of being at node i at the time point t.

Furthermore, we analyzed the statistical significance of scores of each candidate lncRNA. Through comparing the scores of lncRNAs in the network following n iterations of that known MG risk genes shuffling, the statistical significance for rejection of the null hypothesis was determined. In order to strictly maintain the network topological properties, random sampling without replacement was performed when doing random disturbance, and the degree distribution was guaranteed the same between selection seed node and the real. In iterations, the times of each lncRNA score that was higher than the actual value was recorded as m. The statistical significance *P* value for each lncRNA was calculated by the ratio of m and n; in this study, n was set at 1000 times.

### Clinical samples

A total of 31 MG patients were recruited for the sampling of peripheral blood from the Second Affiliated Hospital of Harbin Medical University. All the patients met the diagnostic criteria for MG [27]. None of the patients had undergone hormone treatment for the previous 6 months. Meanwhile, 31 sex- and age-matched healthy subjects with no autoimmune disease were enrolled as the control. Peripheral blood samples obtained from participants were put in tubes containing ethylenediaminetetraacetic acid. Peripheral blood mononuclear cells (PBMCs) were isolated using lymphocyte separation medium. Written informed consent was obtained from all individuals and the study was approved by the Ethics Committee of The Second Affiliated Hospital of Harbin Medical University.

### Real-time PCR analysis

TRIzol reagent (BioTeke, Beijing, China) was applied to isolate total RNA from PBMCs or Jurkat cells. Total RNA was reverse transcribed into cDNA using a RevertAid First Strand cDNA Synthesis kit (Sangon, Shanghai, China) according to the manufacturer’s instructions. Quantitative real-time PCR analysis was performed using Power SYBR Green PCR Master Mix (Solarbio, Beijing, China). The 2^−ΔΔCt^ method was used for the calculation of relative expression level normalized by GAPDH and U6.

### Cell culture

The human embryonic kidney 293T (HEK293T) cells were purchased from Shanghai Zhong Qiao Xin Zhou Biotechnology Co., Ltd (Shanghai, China) and were cultured in Dulbecco’s modified Eagle’s medium (DMEM; Servicebio, China). T cell leukemia line (Jurkat cells) were purchased Procell Life Science & Technology Co., Ltd (Wuhan, China) and were cultured in Roswell Park Memorial Institute 1640 medium (Gibco, USA). All the culture media were supplemented with fetal bovine serum (FBS; Tianhang, Zhejiang, China), penicillin, and streptomycin and incubated at 37 °C in a humidified atmosphere of 5% CO_2_.

### Cell transfection

lncRNA short hairpin RNA (shRNA) of human HCG18, miR-145-5p mimics, miR-145-5p inhibitor, and negative control (NC) were transfected into cells using Lipofectamine 3000 (Invitrogen, USA) following the manufacturer’s protocol. The pool of HCG18 shRNA (shHCG18) contained three shRNAs and three antisense oligonucleotides, which was used to knock down HCG18 expression. The shRNA sequences were as follows: shHCG18-1 target sequence, 5′-AGCTGAAAGTCGACGAAGA-3′; shHCG18-2 target sequence, 5′-CAGCAACTCCTGATGAACA-3′; shHCG18-3 target sequence, 5′-GGTGTAGACAAGACAGCAA-3′; antisense oligonucleotides target sequence-1, 5′-TCTTCGTCGACTTTCAGCT-3′; antisense oligonucleotides targetsequence-2, 5′-TGTTCATCAGGAGTTGCTG-3′; and antisense oligonucleotides target sequence-3, 5′-TTGCTGTCTTGTCTACACC-3′. Total RNA was extracted from transfected cells and subjected to RT-PCR analysis to evaluate transfection efficiency.

### Western blot analysis

Following total protein extraction from Jurkat cells using radioimmunoprecipitation assay solution, protein concentration was determined by bicinchoninic acid protein assay kit (Wanleibio, China). Protein separation was performed using sodium dodecyl sulfate–polyacrylamide gel electrophoresis (SDS-PAGE), and the separated proteins then were transferred onto polyvinylidene fluoride (PVDF) membranes (Millipore, Billerica, MA, USA). PBS containing 5% non-fat milk was used for membrane blocking at room temperature for 1 h. Subsequently, the membrane was incubated with the following primary antibodies overnight at 4 °C: CD28 and β-actin antibodies (Wanleibio), followed by incubation with HRP Goat Anti-Rabbit secondary antibody (IgG, Wanleibio) at room temperature for another 45 min. Signal development was performed using enhanced chemiluminescence (ECL) (Wanleibio) and data was quantified using Gel-Pro-Analyzer software. β-actin was used as the internal control. The experiments were independently repeated in triplicate.

### Luciferase reporter assay

The HCG18 fragment containing miR-145-4p binding sites were synthesized to generate wild type (HCG18-WT) or mutant type (HCG18-MUT). The fragments of HCG18-WT or HCG18-MUT were cloned into the Renilla luciferase gene pmirGLO Luciferase Reporter Vectors (Promega, USA) to generate pmirGLO-HCG18-WT and pmirGLO-HCG18-MUT vectors, respectively. Then, the above vectors were co-transfected together with miR-145-5p mimics or negative control into HEK293T cells using Lipofectamine 3000 (Invitrogen). Luciferase activity was evaluated by a dual-luciferase reporter assay system (Promega, Madison, WI, USA) after 48 h of transfection according to the manufacturer’s protocol.

### Cell proliferation assay

The proliferation of Jurkat cells was determined following the protocol of Cell Counting Kit-8 assay (CCK-8) assay (Wanleibio, China). Each well of a 96-well plate was filled with 0.1 ml suspension containing 4 × 10^3^ cells. Cell culture was performed at 37 °C in a humidified atmosphere of 5% CO_2_. CCK-8 solution was added at 24, 48, 72 and 96 h after the beginning of incubation. The absorbance of each well after incubating at 37 °C for 2 h was measured at 450 nm by using a microplate reader (BIOTEK, USA). The experiments were independently repeated in triplicate.

### Apoptosis analysis

The apoptosis rate of cells was estimated with an annexin V-fluorescein isothiocyanate (FITC)/propidium iodide (PI) apoptosis detection kit (Wanleibio, China) in accordance with the manufacturer’s protocol. Jurkat cells were transferred and cultured in a six-well plate for 48 h and, subsequently, digested with 0.25% trypsin (Gibco). Cells were stained with annexin V-FITC and PI for 15 min in dark, followed by flow cytometry using a NovoCyte Flow Cytometer (Agilent Technologies, Inc, Shanghai, China). Data was analyzed using NovoExpress Flow Cytometry Software (Agilent Technologies, Inc, Shanghai, China). The experiments were independently repeated in triplicate.

### Statistical analysis

GraphPad Prism version 6.0 was used to perform the statistical analysis. Data were presented as the mean ± standard deviation (SD). Student’s t test was performed to analyze the differences between the two groups, while one-way analysis of variance (ANOVA) to analyze that among multiple groups. Correlation analysis was done using Pearson correlation. A p-value < 0.05 (*) was considered statistically significant, and **indicates p < 0.01, and ***p < 0.001, and ****p < 0.0001.

## Results

### Construction of MG-related PPI network and analysis of modules

**Fig. 2:**
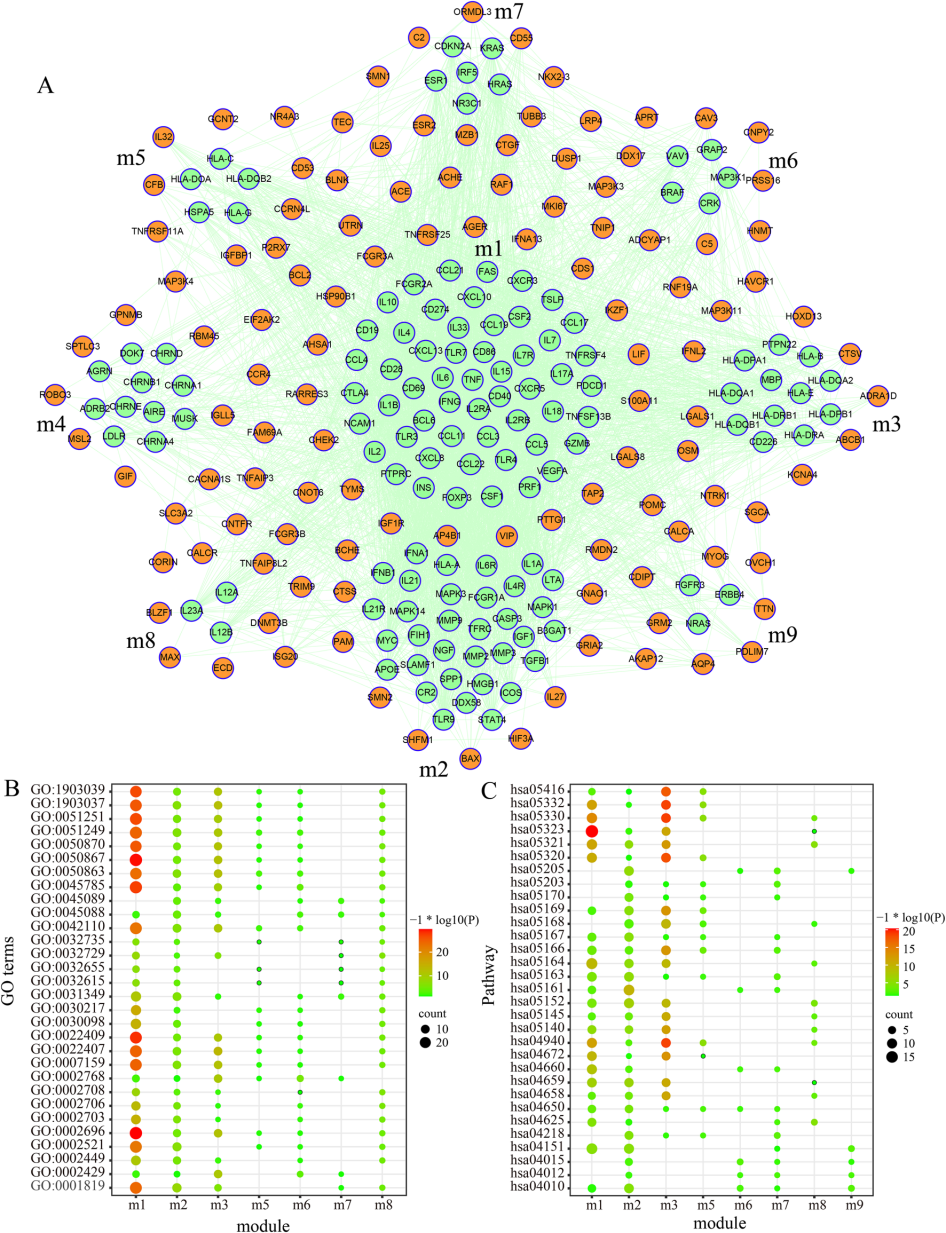
Construction of PPI network and analysis of modules. **a** Construction of PPI network using MG risk genes and identification of modules. All of circles represent MG risk genes, whereas green circles represent genes in the modules. Lines represent interactions between proteins encoded by MG risk gene. **b** GO functional analysis of modules. The degree of enrichment increases from green to red. Larger circles indicate a more significant proportion of MG risk genes in a module among GO function genes. **c** Pathway enrichment analysis of modules. The degree of enrichment increases significantly from green to red. Larger circles indicate a larger proportion of MG risk genes in a module among KEGG pathway genes

It is well known that genes play an important role in the pathogenesis of MG. To clarify the interactions of proteins encoded by MG risk genes, we constructed a PPI network (Fig. 2a), which contained 250 genes and 4211 edges. Furthermore, module analysis was performed to find the key genes shared common properties. A total of 9 modules were identified, including module 1 with 54 genes, module 2 with 33 genes, module 3 with 12 genes, module 4 with 11 genes, module 5 and 6 with 5 genes, module 7 with 6 genes, module 8 and 9 with 3 genes (Fig. 2a). In order to explore the roles of modules in MG, we carried out GO function and KEGG pathway enrichment analysis for each module. As a result, 3257 GO terms and 364 pathways were obtained (p < 0.05) (Additional file 1: Table S1 and Additional file 2: Table S2). GO terms enriched in more than four modules were shown in Fig. 2b and pathways involving more than three modules were shown in Fig. 2c, most of which were relevant to immune or inflammation response. For example, the significant GO terms included T cell differentiation (GO:0030217), T cell activation (GO:0042110), interleukin-12 production (GO:0032615), and positive regulation of cytokine production (GO:0001819). The significant pathway included Th17 cell differentiation (hsa04659), T cell receptor signaling pathway (hsa04660) and natural killer cell mediated cytotoxicity (hsa04650). These findings were consistent with the pathogenesis of MG.

### Construction of the LMMAC network and identification of the key lncRNAs

**Fig. 3:**
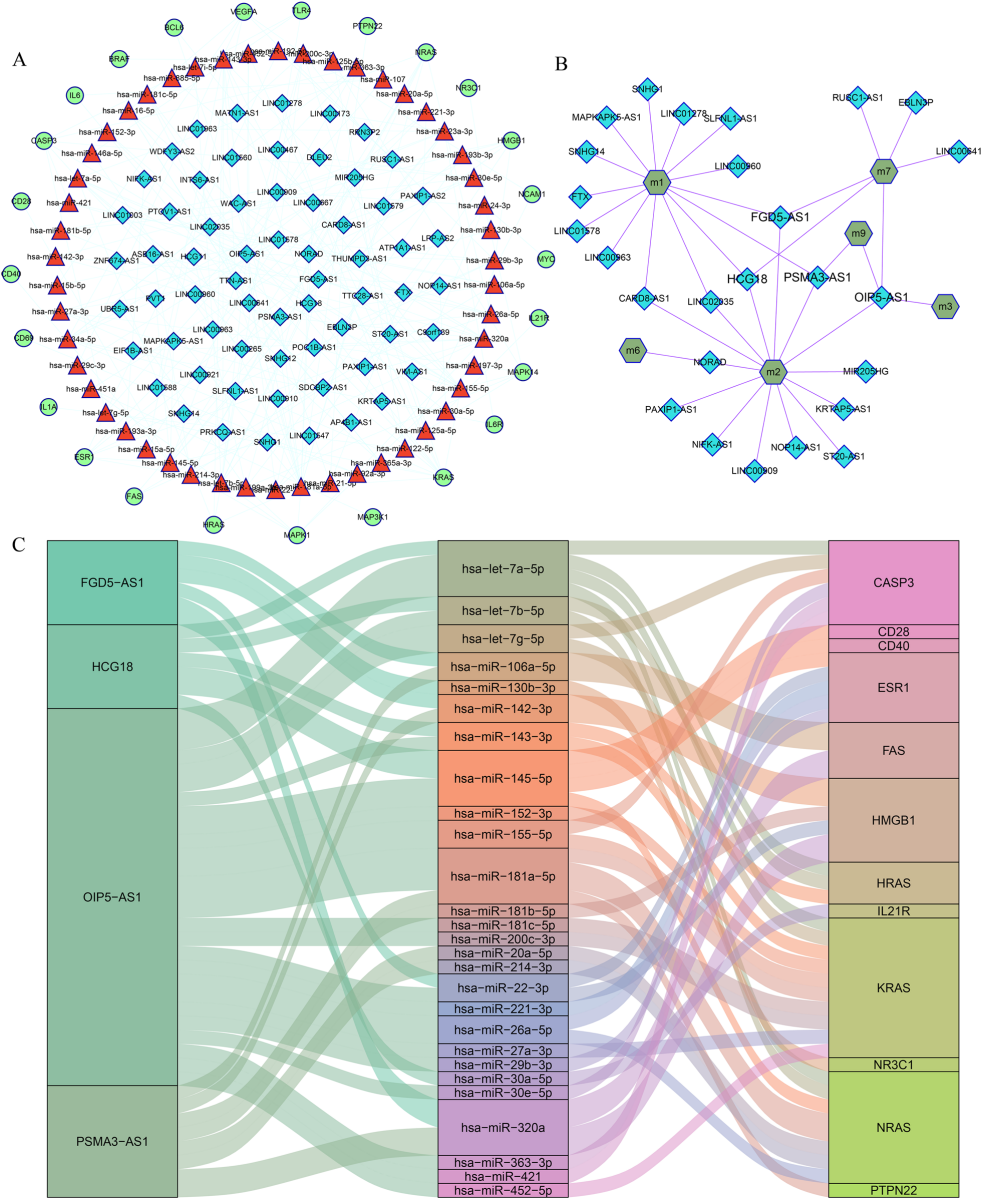
Identification of key candidate lncRNA for MG. **a** The LMMAC network. Green circles represent genes, red triangles represent miRNAs, and blue rhombi represent lncRNAs. Lines represent their regulatory interactions. **b** The lncRNA-module network. Blue rhombi represent lncRNAs, green hexagons represent modules. Lines represent their regulatory interactions. **c** The interactions among four lncRNAs regulating more than two modules, miRNA and genes. The first column denotes lncRNA, the second column denotes miRNA, the third column denotes genes

lncRNAs, acting as ceRNAs, are implicated in various disease. To evaluate the lncRNAs regulation as ceRNAs in MG, a lncRNA-mediated module-associated ceRNA (LMMAC) network was constructed (Fig. 3a). There were 25 genes, 50 miRNAs, 64 lncRNAs and 228 edges in the LMMAC network. Referred to our previous study [28], the number of the primary interaction pairs of lncRNA-miRNA and the secondary interaction pairs of miRNA-gene were counted (Additional file 3: Table S3). For a given lncRNA (i), the number of the primary interaction pairs of lncRNA-miRNA denotes the number of miRNAs connected to lncRNA (i); the secondary interaction pairs of miRNA-gene denote the number of genes connected to the above miRNAs. lncRNA OIP5-AS1 had the highest total number of lncRNA-miRNA and miRNA-gene interaction pairs, which might play an important role in the network.

To further explore the roles of lncRNAs, we first performed RWR algorithm to LMMAC network using MG risk genes in the network as seed genes. After the 1000 times random walk with permutation, we obtained 26 candidate lncRNAs with p < 0.05. As we known that the functions of lncRNAs with high significance scores were high corrected with neighboring seed nodes. So these lncRNAs were considered as the potential regulators of MG, which may involve the pathogenesis of MG. Next, we constructed lncRNA-module network by matching seed genes linked to candidate lncRNAs to modules (Fig. 3b). We found that OIP5-AS1, PSMA3-AS1, FGD5-AS1 and HCG18 could regulate more than two modules, which indicated that they might exert crucially important roles in MG. The interactions among these four lncRNAs, miRNAs, and genes were shown in Fig. 3c. Through reviewing reliable publications, it was found that miR-145-5p/CD28 regulation pairs had reportedly been validated in MG [29]. CD28, as a positive regulator of T cell proliferation and cytokine, could promote T cell reactivity in MG [30]. In our study, HCG18 was the only one lncRNA potentially competing with CD28, which had been reported to be relevant to immune cell infiltration in cancer [31]. Therefore, we mainly focused on HCG18/miR-145-5p/CD28 interaction for further experimental verification.

### lncRNA HCG18 is upregulated in MG patients and is a target of miR-145-5p

**Fig. 4:**
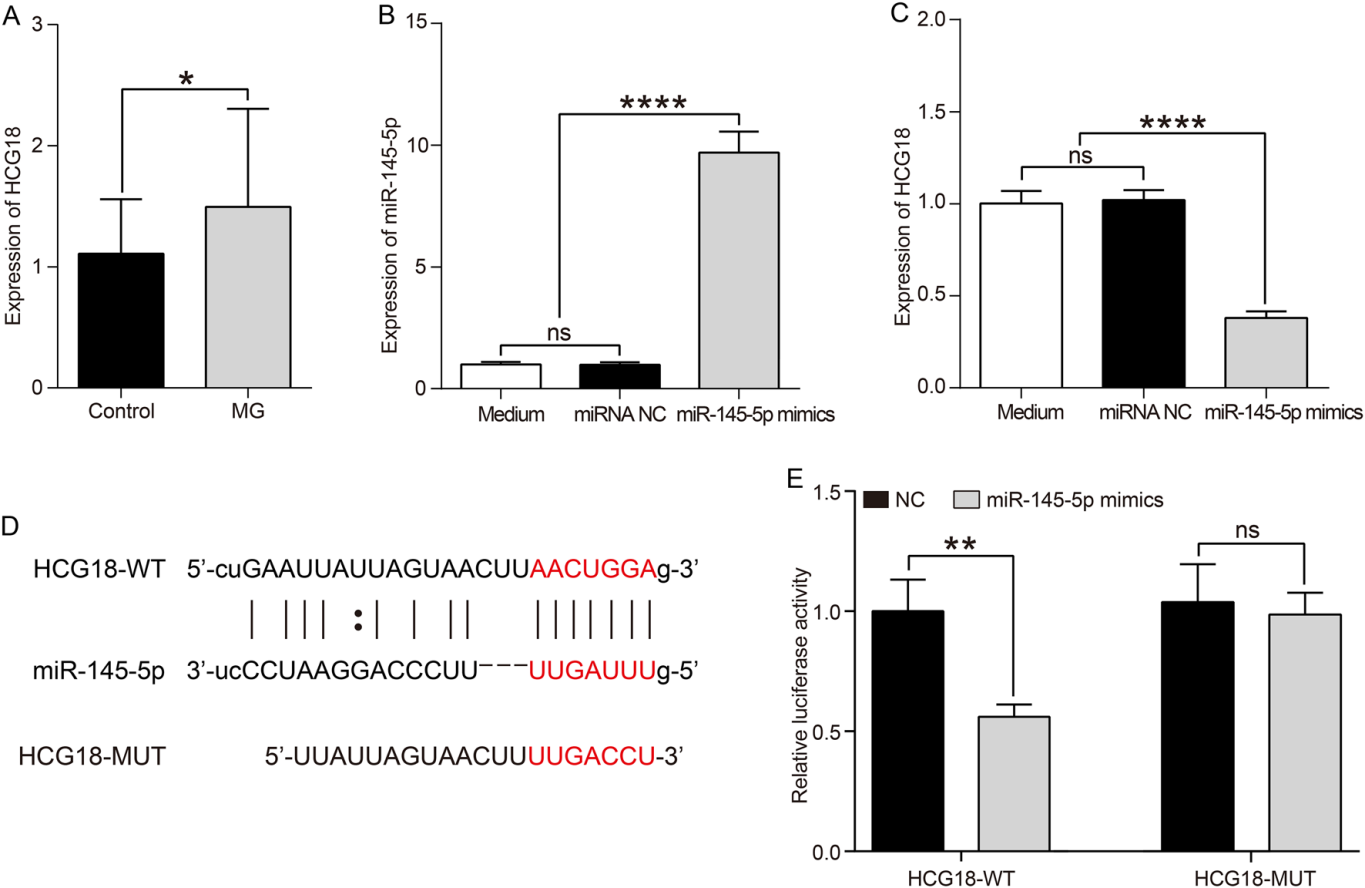
Upregulation of HCG18 is a target of miR-145-5p in MG. **a** The expression of HCG18 was examined in MG patients and control subjects by RT-PCR. **b** Transfection efficiency of miR-145-5p mimics was determined by RT-PCR. **c** The relative expression level of HCG18 after transfecting with miRNA NC or let-7c-5p mimics into Jurkat cells was measured using RT-PCR. **d** The putative miR-145-5p binding sequence of the wild type (WT) and mutation (MUT) sequence of HCG18. **e** The luciferase reporter plasmid containing HCG18-WT or HCG18-MUT was co-transfected with miR-145-5p mimics or miRNA NC into 293 T cells. The ratio of firefly/renilla activities represented luciferase activitie. The experiment was repeated at least three times, and data are presented as the mean ± SD, *p < 0.05, **p < 0.01, ****p < 0.0001

The expression of lncRNA HCG18 was examined by RT-PCR in PBMCs from patients with MG and control subjects. HCG18 was upregulated in MG patients compared with controls (p = 0.0251, Fig. 4a). It was found that HCG18 sequence contains a potential miR-145-5p binding region through bioinformatics analysis. miR-145-5p was downregulated in PBMCs of MG patients according to a previous study [29]. To investigate the relationship between miR-145-5p and HCG18, miR-145-5p mimics were transfected into the Jurkat cells. RT-PCR was performed to determine the transfection efficiency of miR-145-5p mimics (p < 0.0001, Fig. 4b). HCG18 expression was significantly suppressed by overexpression of miR-145-5p in Jurkat cells (p < 0.0001, Fig. 4c). We further constructed luciferase reporter vectors of HCG18-wild type (WT) and HCG18-mutated type (MUT) to confirm the direct interaction between HCG18 and miR-145-5p (Fig. 4d). Then, HCG18-WT or HCG18-MUT and miR-145-5p mimics or negative control (NC) were co-transfected into 293 T cells. The dual-luciferase reporter assay indicated that miR-145-5p mimics inhibited the luciferase activity of HCG18-WT but had no effect on that of HCG18-MUT (p < 0.01, Fig. 4e). These results illustrated that HCG18 is a target of miR-145-5p.

### HCG18 promotes CD28 expression by sponging miR-145-5p

**Fig. 5:**
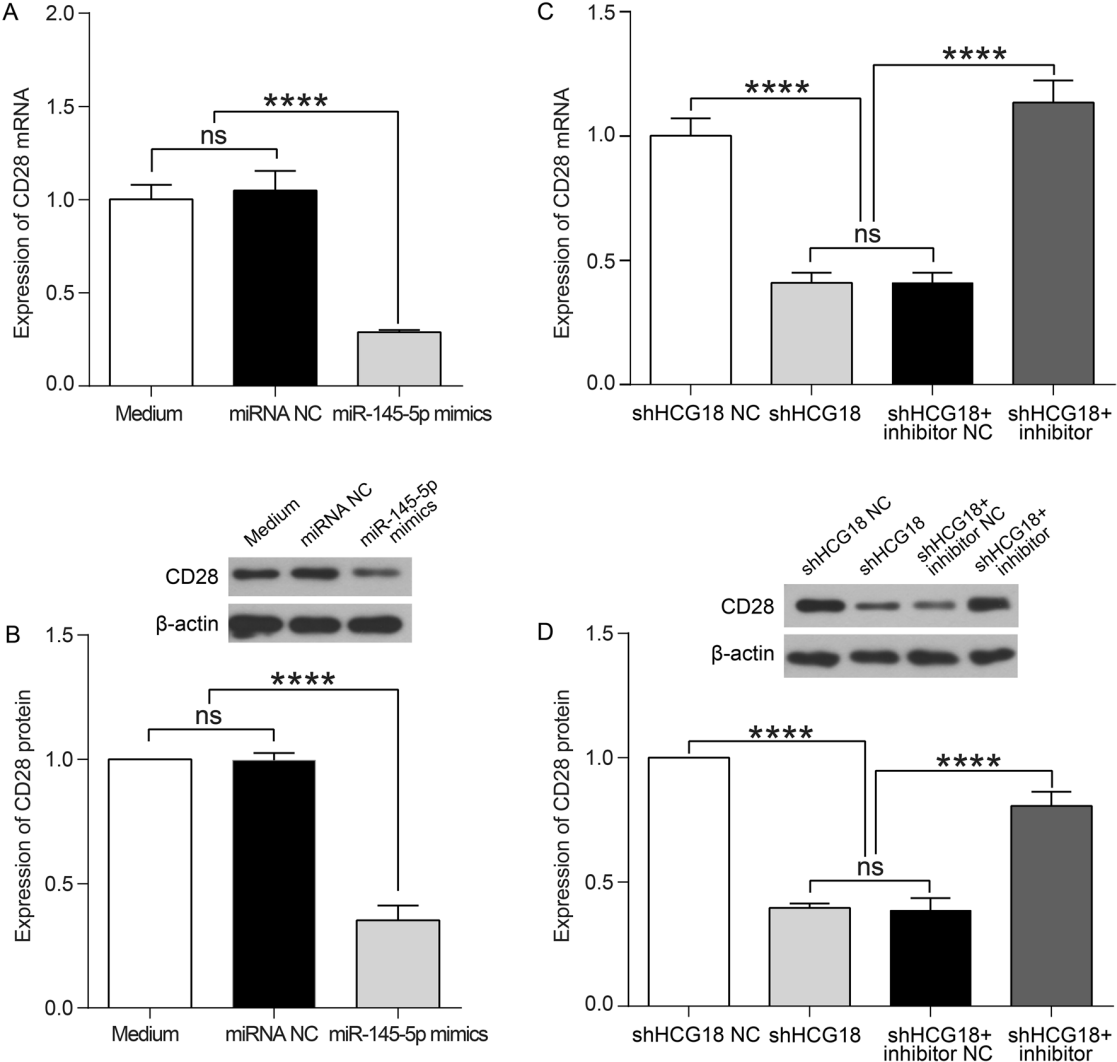
HCG18 regulates CD28 expression by sponging miR-145-5p in a ceRNA manner. **a** Expression levels of CD28 mRNA were examined by RT-PCR after transfection with negative control or miR-145-5p mimics in Jurkat cells. **b** Expression levels of CD28 protein were examined by western blotting after transfection with negative control or miR-145-5p mimics in Jurkat cells. **c** Expression levels of CD28 mRNA were examined by RT-PCR analysis after transfection with negative control, shHCG18, and shHCG18 + miR-145-5p inhibitor in Jurkat cells. **d** Expression levels of CD28 protein were examined by western blotting after transfection with negative control, shHCG18 and shHCG18 + miR-145-5p inhibitor in Jurkat cells. The experiment was repeated at least three times, and data are presented as the mean ± SD, ****p < 0.0001

As known, MG is a T-cell-associated autoimmune disease, while costimulatory molecules are crucial for the activation of T cells [32]. A study has reported that there is negative correlation a between miR-145-5p and CD28 mRNA level in MG, and that miR-145-5p inhibit CD28 expression by directly targeting the 3′-UTR of the CD28 mRNA in CD4^+^ T cell [29]. Therefore, an experiment was designed to explore whether HCG18 could regulate CD28 expression by sponging miR-145-5p. We first determined the expression level of CD28 mRNA and protein after transfecting miR-145-5p mimics or NC into Jurkat cells. We found that both mRNA and protein level of CD28 were reduced by overexpressed miR-145-5p (p < 0.0001, Fig. 5a, b), which is consistent with previous study [29].

To verify whether HCG18 regulates the expression of CD28 via targeting miR-145-5p, Jurkat cells were transfected with shHCG18 NC, shHCG18, and shHCG18 in combination with miR-145-5p inhibitor NC or inhibitor. We then detected the expression level of CD28 mRNA and protein. The results showed that knockdown of HCG18 restrained the CD28 mRNA and protein expression levels in Jurkat cells, while miR-145-5p inhibitor blocked the reduction of CD28 expression induced by HCG18 suppression (p < 0.0001, Fig. 5c, d). The findings suggested that HCG18 regulated CD28 expression by sponging miR-145-5p.

### HCG18 inhibits apoptosis and promotes proliferation by sponging miR-145-5p in Jurkat cells

**Fig. 6:**
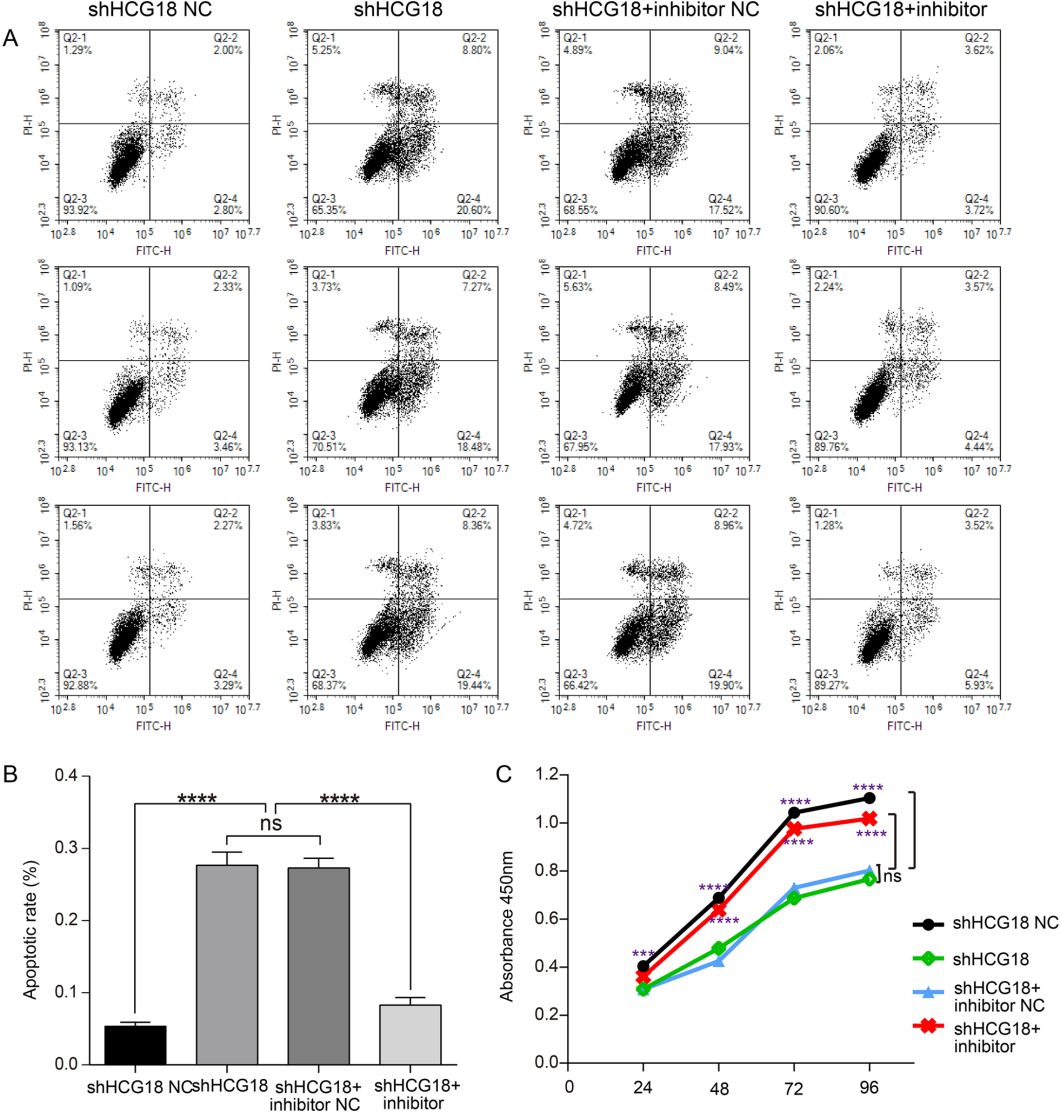
HCG18 inhibits cell apoptosis and promotes cell proliferation by targeting miR-145-5p. **a**, **b** Flow cytometric analysis was performed using cells stained with Annexin-V-FITC/PI after transfecting negative control, shHCG18, or shHCG18 + miR-145-5p inhibitor into Jurkat cells. **c** The proliferation of Jurkat cells was analysed using CCK-8 assays by transfecting negative control, shHCG18, or shHCG18 + miR-145-5p inhibitor into Jurkat cells. The experiment was repeated at least three times, and data are presented as the mean ± SD, ***p < 0.001, ****p < 0.0001

MG is a T-cell-dependent nervous system autoimmune disease. The activation and proliferation of T cells exert a significant role in the pathogenesis of MG. Hence, an experiment was designed to validate whether HCG18 affects the apoptosis and proliferation of T cells by sponging miR-145-5p in MG. According to the previous studies [28, 33], Jurkat cells were used for the functional verification and were transfected with shHCG18 NC, shHCG18, and shHCG18 in combination with miR-145-5p inhibitor NC or inhibitor. Then, the rates of apoptosis and proliferation were determined by flow cytometry and CCK-8 assay, respectively. It was observed that the rate of apoptosis in Jurkat cells increased following transfection with shHCG18, while the addition of miR-145-5p inhibitor significantly weakened the above trend (p < 0.0001, Fig. 6a, b). Meanwhile, CCK-8 assay showed that the proliferation of Jurkat cells was notably repressed in the shHCG18 group compared with the negative control group, and the effects was offset by miR-145-5p inhibitor (p < 0.001, Fig. 6c). These findings demonstrate that HCG18 inhibit apoptosis and promote proliferation of Jurkat cells by targeting mR-145-5p, which might be involved in the immunological pathogenesis of MG.

## Discussion

MG is a complex autoimmune disease of nervous system, the pathogenesis of which has not been fully illuminated. Genes expressed by immune cells, such as interleukin (IL)-2, interferon (IFN)-γ, and tumor necrosis factor (TNF)-α, are crucial regulators of the occurrence and development of MG [34]. Increasing evidence has elaborated that lncRNAs play important roles in the pathogenesis of autoimmune diseases [35]. Especially, lncRNAs acting as ceRNAs could participate in regulation of gene expression, which provided a novel insight into improving our understanding of the precise molecular mechanism of MG.

To date, the roles of ceRNA regulatory mechanism have been validated in various diseases. For example, Zhang et al. [36] identified a potential regulatory axis of RP11-1094M14.8/miR-1269a/CXCL9 in gastric cancer with different degrees of immune cell infiltration by constructing a lncRNA-mediated ceRNA network. Wang et al. found that Linc00284 promoted lung cancer progression by acting as a ceRNA of miR-205-3p to regulate c-Met expression [37]. However, most studies involving ceRNA network and regulatory mechanism focused on cancer field, only a few on autoimmune disease. Therefore, there is an urgent need to explore the ceRNA regulatory mechanism in MG. Additionally, functional modules in a network represent nodes shared common properties, which are useful to infer the function of unknown nodes. Accordingly, we first constructed a PPI network based on MG risk genes and identified 9 modules. The results of functional annotation analysis show that modules were closely related to T cells, which is consistent with MG pathogenesis. Then, an LMMAC network was constructed based on ceRNA theory using a comprehensive approach, which was composed of 25 genes, 50 miRNA, 64 lncRNAs. Next, we performed RWR algorithm to the LMMAC network and obtained 26 candidate lncRNAs that were considered as the potential regulators of MG. Of note, OIP5-AS1, PSMA3-AS1, FGD5-AS1 and HCG18 could modulate more than two modules, which suggested these lncRNAs concerned multiple biological pathways and played critical roles in pathogenesis of MG.

Furthermore, we found that the expression of miRNA-gene pair regulated by HCG18 has been verified in MG. In recent years, growing evidence illustrates that HCG18 as a ceRNA is involved in several diseases. For example, Zou et al. [38] confirmed that HCG18 serves as a ceRNA to promote the proliferation and migration of hepatocellular carcinoma via competitively binding miR-214-3p/CENPM. Li et al. showed that HCG18 contributes to nasopharyngeal carcinoma development by sponging miR-140 to regulated the expression of CCND1 [39]. Importantly, a recent study revealed that HCG18 as a ceRNA affected immune processes. In Diabetic peripheral neuropathy rats, HCG18 promotes M1 macrophage polarization by regulating the miR-146a/TRAF6 axis [40]. However, the underlying regulatory mechanism of HCG18 in MG remains unclear. In the present study, the expression of HCG18 was increased in MG patients. Functionally, it was found that knockdown of HCG18 promotes apoptosis and inhibits cell proliferation in Jurkat cells. These finding suggest that HCG18 might participate in the immunological pathogenesis of MG.

HCG18 was predicted to be a target of miR-145-5p through bioinformatics analysis. It has been reported that miR-145-5p expression was decreased in PBMCs from MG patients and CD28 was a target of miR-145-5p [29]. Importantly, miR-145-5p overexpression suppressed CD28 expression and proliferative response in CD4^+^ T cells. In our work, we also found that upregulation of miR-145-5p inhibited CD28 mRNA and protein expression in Jurkat cells, which were consistent with the results of previous study [29]. Therefore, we hypothesized that HCG18 served as a ceRNA to regulate miR-145-5p/CD28 axis in MG. It was found that transfection of miR-145-5p mimics notably decreased the expression level of HCG18 in Jurkat cells. Meanwhile, results of luciferase reporter assays showed that HCG18 is a direct target of miR-145-5p. Knockdown of HCG18 repressed the CD28 mRNA and protein levels, but these effects were offset by co-transfecting shHCG18 and miR-145-5p inhibitor into Jurkat cells. In addition, the functional experiment suggests that HCG18 knockdown promoted cell apoptosis and inhibited cell proliferation, whereas co-transfecting shHCG18 and miR-145-5p inhibitor into Jurkat cells reversed the above effects. Our results show that HCG18 severs as a ceRNA to regulate CD28 expression by competitively binding miR-145-5p. T cells play a role in assisting B cell humoral immunity, which are essential for both the production of antibodies and the maintenance of the germinal centers. In other word, pathogenic autoantibodies in MG form as a result of interaction of T cells and B cells. CD28, as a member of costimulatory molecules, is essential for the activation of T cells. Thus, the present study suggested that HCG18 might promote the pathological progression of MG by regulating miR-145-5p/CD28 axis.

Though the pathogenesis of MG is gradually being uncovered, the treatment of MG remains a challenge. Given the complex pathogenesis of MG and the side effects of drugs, no one treatment therapy is best for all MG patients. In the past few years, we have tried to adopt several different methods, such as constructing miRNA-regulated drug-pathway network [41] and TF-miRNA-gene feed-forward loop network [42], to screen candidate treatment drugs for MG. Recently, the combination of emerging nanotechnologies and material sciences has promising applications in multiple fields, such as nanotechnology in food and agriculture [43], nano-antioxidants in environmental-related oxidative damages [44] and nanotechnology on drug delivery in autoimmune diseases [45]. Targeted delivery of drugs to the specific tissues might be the most prominent feature of nanotechnology in biomedicine. The implementation of nanomedicine not only offers enhanced drug solubility and reduced off-target adverse effects, but also provides novel therapy approaches in clinical practice [5]. So, well-designed experiments should be conducted in the future to search appropriate nanomaterials for MG, and to develop new therapy strategy for MG patients.

## Conclusion

In the present study, we for the first time constructed a lncRNA-mediate module-associated ceRNA network. A novel regulatory mechanism on lncRNA HCG18 was uncovered in MG, which regulated the expression of CD28 by targeting miR-145-5p. Our results suggested that construction of LMMAC network provided a global view for the mechanisms of ceRNA regulation and a significant background for the molecular mechanism studies of MG. Our study will contribute to a deeper understanding of the regulatory mechanism of MG and will potentially provide candidate lncRNAs as biomarkers for diagnosis and therapies in MG.

## Supplementary Information

**Additional file 1: Table S1.** The results of GO functional enrichment analysis of 9 modules.

**Additional file 2: Table S2.** The results of pathway functional enrichment analysis of 9 modules.

**Additional file 3: Table S3.** The number of the primary interaction pairs of lncRNA-miRNA and the secondary interaction pairs of miRNA-gene.

## Data Availability

The datasets used and/or analysed during the current study are available from the corresponding author on reasonable request.

## Abbreviations

MG: Myasthenia gravis
lncRNAs: Long non-coding RNAs
ceRNAs: Competing endogenous RNAs
PBMCs: Peripheral blood mononuclear cells
RWR: Random walk with restart
GO: Gene ontology
PPI: Protein–protein-interaction
